# An agriculture and health inter-sectorial research process to reduce hazardous pesticide health impacts among smallholder farmers in the Andes

**DOI:** 10.1186/1472-698X-11-S2-S6

**Published:** 2011-11-08

**Authors:** Donald C Cole, Fadya Orozco T, Willy Pradel, Jovanny Suquillo, Xavier Mera, Aura Chacon, Gordon Prain, Susitha Wanigaratne, Jessica Leah

**Affiliations:** 1 Dalla Lana School of Public Health, University of Toronto, Toronto, Ontario, Canada, M5T 3M7; 2 Federal University of Bahia, Institute of Collective Health, Brazil, based in Ecuador; 3 International Potato Center, Lima, Peru; 4 National Research Institute of Agriculture and Livestock, Carchi, Ecuador; 5 International Potato Center, Pillaro, Ecuador; 6 Cancer Care Ontario, Canada; 7 City of Toronto, Ontario, Canada

## Abstract

**Background:**

The use of highly hazardous pesticides by smallholder farmers constitutes a classic trans-sectoral ‘wicked problem’. We share our program of research in potato and vegetable farming communities in the Andean highlands, working with partners from multiple sectors to confront this problem over several projects.

**Methods:**

We engaged in iterative cycles of mixed methods research around particular questions, actions relevant to stakeholders, new proposal formulation and implementation followed by evaluation of impacts. Capacity building occurred among farmers, technical personnel, and students from multiple disciplines. Involvement of research users occurred throughout: women and men farmers, non-governmental development organizations, Ministries of Health and Agriculture, and, in Ecuador, the National Council on Social Participation.

**Results:**

Pesticide poisonings were more widespread than existing passive surveillance systems would suggest. More diversified, moderately developed agricultural systems had lower pesticide use and better child nutrition. Greater understanding among women of crop management options and more equal household gender relations were associated with reduced farm pesticide use and household pesticide exposure. Involvement in more organic agriculture was associated with greater household food security and food sovereignty. Markets for safer produce supported efforts by smallholder farmers to reduce hazardous pesticide use.

Participatory interventions included: promoting greater access to alternative methods and inputs in a store co-sponsored by the municipality; producing less harmful inputs such as compost by women farmers; strengthening farmer organizations around healthier and more sustainable agriculture; marketing safer produce among social sectors; empowering farmers to act as social monitors; and using social monitoring results to inform decision makers. Uptake by policy makers has included: the Ecuadorian Ministry of Health rolling out pesticide poisoning surveillance modeled on our system; the Ecuadorian Association of Municipalities holding a national virtual forum on healthier agriculture; and the Ecuadorian Ministry of Agriculture promulgating restrictions on highly hazardous pesticides in June 2010.

**Conclusion:**

Work with multiple actors is needed to shift agriculture towards greater sustainability and human health, particularly for vulnerable smallholders.

## Background

The use of highly hazardous pesticides by smallholder farmers, especially in lower and middle income countries (LMICs) constitutes a classic trans-sectoral ‘wicked problem’. Wicked problems are those where good social solutions are difficult to achieve because of differences among stakeholders in framing, understanding and responding to the problem [1]. Smallholder farmers, those with land size of approximately 0.2 to 2.0 hectares and net incomes of hundreds of dollars per hectare [2]) tend to be poorer than rural populations overall, yet constitute one of the major population groups in many LMICs - 70 million in Latin America alone [3]. For many smallholder farmers, cheap, hazardous pesticides have been effective and profitable in the short term [4] thereby guaranteeing farm production, wages for occasional contract labourers, and family survival [2].

Pesticides most commonly used by smallholder farmers in Ecuador are those categorized by the WHO [5] as extremely (Ia), highly (Ib) or moderately toxic (II), mostly organophosphorus and carbamate compounds. Limited understanding of pesticide container labels, lack of knowledge of alternative approaches, and difficulties in obtaining adequate personal protective equipment occur because of lack of interest, resources or commitment among governmental and industry actors [6]. The consequences in northern Ecuador have been acute pesticide poisonings at rates among the highest recorded globally [7] and longer term neurotoxic effects among the majority of smallholder farm adult populations [4]. Further, neurobehavioral function among Carchi, Ecuador farmers dropped from a mean score of 6.9/10 (10 is excellent performance) to 4.4/10 over the decade of the 90’s [unpublished data].

Agricultural sector investment in LMICs has been strongly oriented towards medium to large scale commercial agriculture [3]. Government actions to reduce pesticide-related externalities on human health and the environment have been minimal. Nevertheless, health education and alternative agricultural practices can achieve improvements in agricultural productivity (similar yields with fewer input costs) and human health (better neurobehavioral function) [8].

In this paper, we summarize action-research processes among potato and vegetable farming communities in highland Ecuador undertaken through three Canadian funded projects: *Ecosalud [EcoHealth] II* (2005-2008), *Hortisana [Healthy Horticulture]* (2007-2010), and *Gobernanza con Capital Social [Social Capital and Accountability]* (2008-2011). Partnerships underpinning these projects involved core Andean-based organizations (the International Potato Center, referred to by its Spanish acronym CIP, in Peru and Ecuador, and the National Institute of Agricultural and Livestock Research in Ecuador, referred to by its Spanish acronym INIAP) linked with both external collaborators - primarily with the University of Toronto and affiliated institutions- and internal, within-country collaborators. The latter have included nongovernmental development organizations, provincial ministries of health and agriculture, organizations of independent smallholder family farmers, agricultural marketing organizations, research institutes and municipalities (see fuller description of partnerships below and [9]). Our overall goal has been to tackle the complex drivers associated with the use of highly hazardous pesticides among smallholder farmers, aiming for the improvement of human health within sustainable agro-ecosystems.

## Research – action methods

The development of our research program has been an iterative process: starting with particular questions, and working with stakeholders to obtain results in an assessment phase. Research teams included health (DCC, FOT, SW, JL), bio-physical/natural (JS, XM, AC), social (WP, GP) scientists and practitioners. Young professionals-researchers in training have joined us in a trans-disciplinary approach [10]. Mixed methods (surveys, key informant interviews, focus groups, participatory appraisals and document reviews) were used at the beginning of Ecosalud II, in Ecuador, and HortiSana in three Andean countries. Initial assessments helped us to identify three immediate challenges: 1) Lack of information on pesticides and alternatives; 2) Limited markets both for less toxic inputs for farmers and safer produce for consumers; and 3) Limited farmer organization to strengthen implementation of sustainable agriculture alternatives or citizen participation to demand social accountability and enforcement of existing regulations. Each of the immediate challenges led us to focus intervention activity with evaluation, in keeping with public health intervention research approaches [11]. Implementation of interventions often left gaps which new initiatives with stakeholders were then designed to address.

## Results and outcomes

### General

The geographic areas of our research program were highly diverse, with a wide variety of production systems and education, poverty, ethnicity and gender relations. In keeping with the observations of rural sociologists [14], we found that smallholder family farms were highly heterogeneous in production orientation. In Ecosalud II, children under five on either highly commercial or subsistence farms were found to have poorer nutrition than intermediate farms [2]. Commercial farm children had a higher prevalence of micronutrient intake deficiencies most likely due to a less diverse and nutrient poor diet among commercial farm families who were primarily purchasing cheap but less nutritious foods e.g. pasta. Across HortiSana metropolitan regions, we found that household food security [13] was associated with better housing, greater participation in agricultural associations, larger area of irrigated land, and greater satisfaction with health status.

### Lack of information

In the Ecosalud II project, knowledge of pesticide-related hazards and practices to reduce exposure was limited and varied by gender. At baseline, women’s greater participation in farm decision-making and a more equal allocation of production and household roles between genders was associated with less use of highly hazardous pesticides on the farm, particularly those farms with less of a commercial orientation [14]. Informed by observations in community meetings and during focus groups, we attributed this association to women’s greater concerns with the health of their families, both of their husbands-male partners and of their children. Greater women’s voice in couples seemed to be associated with greater influence of these health concerns on smallholder production practices. Nevertheless, understanding of the degree of hazard based on pesticide container label colors and symbols was lower for women at baseline (within household gender differences significant at p<0.05 using t-test) [14].

The Ecosalud II team addressed this challenge through a health promotion intervention guided by socio-ecological conceptual frameworks which encompass a range of theories on behaviour change at multiple levels [11]. Our objectives were: a) to encourage farmer reflection on their farming practices and the health and environmental consequences of them; b) to improve all farm household members’ accessibility to information on handling and use of pesticides and alternative agricultural production methods; and c) to support changes in practices. We used various educational techniques including role modeling and co-discovery in farmer field schools, field days, community theatre, puppet shows with children, and interactive sessions with women and men in 24 communities in three Ecuadorian provinces [15]. Although both partners improved their knowledge at follow-up, women improved less: mean score of 1.2/10 increased to 3.4/10 for women, paired t-test t=10.38 p<0.0001 compared to 2.6 to 5.3 for men, paired t-test t= 10.4 p<.0001. [15]. We attributed the latter difference to the lesser participation of women in our interventions, particularly those more agriculturally oriented. On the positive side, among women participating in HortiSana, we heard stories of substantial increases in the diversity of vegetables which women farmers were producing and which their families, particularly children, were consuming [Xavier Mera, unpublished report].

### Limited markets

The Ecosalud II team found that farmers did not have access to inputs such as insect traps or less hazardous pesticides. We worked with a farmers’ organization and a local municipality in co-sponsoring a store to provide such inputs, along with advice on how best to use them. In the HortiSana project, a women’s agricultural association expanded their composting facility to provide both themselves and others with a key input for vegetable production. In Peru, farmers expressed concerns about climate change affecting the diversity of agricultural products they produced. However, they thought that the threats to their food security were due primarily to fluctuating returns for their crops while purchased food prices were increasing. In both Ecuador and Peru, the HortiSana team found that consumer demand for organic or “healthy” (*sana*) products was increasing as was interest in indigenous foods. Direct-marketing arrangements like a local organic market (*Bioferia*) in Peru or an Agricultural Association-sponsored store in Ecuador facilitated the formation of relationships of trust between producers and consumers [Xavier Mera, unpublished report]. The arrangements provided greater financial returns to farmers and quality assurance to consumers. Feedback up the value chain from consumers indicated that they sought better quality, greater variety of vegetables, more consistent availability and larger volumes [Jennifer Lomas, unpublished report].

### Limited citizen participation

In the social accountability project, the team found substantial gaps in the application of existing regulations for, or international conventions on, adequate use and management of pesticides in small scale agriculture [16]. Social and political institutions were found to be fragmented, with an unclear delineation of responsibilities, making it hard to move forward with joint actions.

Grounded in theories of social capital [17], the team has focused on the promotion of a set of “Farmers' Rights” [6] to command the attention of political decision-makers. Intervention activities have included provision of information on Farmers’ Rights, invitations to farmer-volunteers to undergo training on these rights, and encouragement of involvement in similar citizen engagement and social action processes. Mobilized citizens have pushed for development and implementation of public policies which preserve the health of smallholder farmers and their families. They have set up a surveillance system to track implementation of existing regulations and of people’s understanding of them [16].

## Partnerships

Given the diversity of organizations involved, the multiple components or steps involved in our research-development process and the different levels of action (local, regional, national), our partnerships have inevitably been complex and dynamic [18,19]. Though complementary in the overall program of work, we will focus on the research, implementation and knowledge use partnerships in turn.

Research partnership has been facilitated by a longstanding Associate Scientist position held by the key Canadian researcher (DCC) with CIP, where the lead social scientist is located (GP). In Ecuador, a formal relationship of the key Ecuadorian health scientist (FOT) with INIAP and a mentoring relationship between the Canadian and Ecuadorian leads has helped immensely. Each lead contributes to joint conceptualization, design, and obtaining funding for the research, oversee its implementation and takes primary responsibility for analysis and reporting to the broader scientific community. These core research collaborations have been complemented through multi-disciplinary agricultural research alliances with national and international scientists in CIP and other Ecuadorian and Peruvian organizations. Decisions have been primarily made in country by collaborative research-action teams, complemented by supporting virtual dialogue (email, Skype) among Canadian and Andean colleagues.

Implementation partnerships involved dynamic shifts in the core collaborations among organizations strongly linked with Andean institutions. Foremost among these were farmers' organizations. The common role of the project teams has been to strengthen existing organizations, though the HortiSana team has also worked with farmer innovators to facilitate organization of groups of organic farmers in municipalities where no such organizations existed. Representatives of nongovernmental organizations (NGOs) which regularly work with farmers and municipal technicians, and who increasingly perform development roles in their jurisdictions, played key roles during implementation. To formalize links between, for example CIP or INIAP and NGOs, memoranda of understanding have been elaborated, specifying in detail the nature of the collaboration, resource flows, counterpart support and mutual objectives. NGOs have also worked with a wide range of stakeholders, often bringing them together in “platforms”, and social spaces where discussion, coordination, project development and learning from research results could occur. An example is the “Platform for Inter institutional Dialogue to Work towards Sustainable Production in the Mantaro Valley” in Peru, in which the idea of a “bioferia” in Chupuro, Peru was developed into a successful marketing and health education event [20].

A third kind of partnerships has involved knowledge use by mainly policy actors, usually at higher levels of government, who can position the activities and results of the intervention-research process. Relationships with these political actors have been more complex, often taking the form of shifting alliances among a wider range of social actors. For example, in policy and regulatory initiatives, such as the prohibition of extremely and highly hazardous pesticides in Ecuador [21], much work is required by multiple civil society actors working with sympathetic government actors to undertake effective implementation.

## Challenges and successes

The fundamental challenge of persistent and deepening inequities in determinants of health in the Andes [22] has both historical roots and present day causes in the functioning of the current model of globalization [23]. Our research-action process sought to address these underlying causes, particularly those related to agricultural production, but was constrained by them. During EcoSalud II interventions, vertical approaches to community leadership excluded broader social participation and limited some community members’ access to education programs on pesticides, crops and health [15]. We noted greater change in communities with fewer unsatisfied basic needs and among households with greater household assets [Cole, Orozco, et al., unpublished data]. In Hortisana interventions in Peru, those who tended to remain as members of associations had relatively more assets than those who initially participated in the Field Schools [Pradel, unpublished paper]. On the other hand, the existing organizations with whom Hortisana worked in Ecuador tended to include producers with fewer resources, suggesting that interventions in support of these associations were able to confront inequalities.

Trans-disciplinary research for development also offered multiple challenges [25] as did inter-organizational collaboration. Collaboration was initially difficult because of perceived competition for resources, for example between established local NGOs and newly active international organizations. Subsequently diverging priorities, often driven by changing funding opportunities, created tensions. To counteract the potential of competitive fragmentation between disciplines and organizations, major efforts were devoted to openness, transparency and dialogue in the second type of partnerships, actively promoting construction of collaborative methods and strategies based on a set of common objectives. Strong negotiating skills were needed to reinforce social networks, to incorporate results of ongoing research, to inform interventions, and to generate new research questions and projects for the benefit of smallholder households. Actualizing goals of equity and well- being, such as those contained in Ecuadorian legislative [26] and policy instruments, requires ongoing social participation of smallholder farmers in both research action processes, such as those described here and broader social movements which they can inform.

## List of abbreviations used

CIP: International Potato Center; IIN: Institute for Research in Nutrition, Peru; INIAP: National Institute for Agriculture and Livestock Research in Ecuador; LMIC: lower and middle income countries; NGO: non-governmental organization; WHO: World Health Organization.

## Competing interests

The authors declare that they have no competing interests, other than those identifiable through their institutional affiliations.

## Authors' contributions

DCC conceived the article, co-designed the research for two projects and participated in their analysis and interpretation, co- wrote the initial draft and revised the manuscript.

FO is co-researcher and coordinator for Ecosalud II project; conceived, designed, and is the main researcher of the Gobernanza Project, is co-writer of the article and revised the manuscript.

WP was involved in the coordination and evaluation of the initial draft of the HortiSana project and revised the manuscript.

JS, AC, XM and SW all were involved in implementation of different projects.

GP co-conceived one project, has co-lead implementation of the same and made revisions to the manuscript.

JL analyzed and interpreted data for the HortiSana project; and revised the manuscript.

All authors read and approved the final manuscript.
